# The Effect of Recombinant Tags on *Citrus paradisi* Flavonol-Specific 3-O Glucosyltransferase Activity

**DOI:** 10.3390/plants9030402

**Published:** 2020-03-24

**Authors:** Aaron S. Birchfield, Cecilia A. McIntosh

**Affiliations:** Department of Biological Sciences, P.O. Box 70703, East Tennessee State University, Johnson City, TN 37614, USA; birchfieldas@etsu.edu

**Keywords:** glucosyltransferase, recombinant tags, His-tag, flavonoids, flavonol, grapefruit, C-terminal recombinant tags, pH optima, reaction kinetics

## Abstract

Recombinant tags are used extensively in protein expression systems to allow purification through IMAC (Immobilized Metal Affinity Chromatography), identification through Western blot, and to facilitate crystal formation for structural analysis. While widely used, their role in enzyme characterization has raised concerns with respect to potential impact on activity. In this study, a flavonol-specific 3-O glucosyltransferase (Cp3GT) from grapefruit (*Citrus paradisi*) was expressed in *Pichia pastoris*, and was assayed in its untagged form and with a C-terminal c-myc/6x His tag under various conditions to determine the effect of tags. Prior characterization of pH optima for Cp3GT obtained through expression in *Escherichia coli*, containing an N-terminal thioredoxin/6x His tag, indicated an optimal pH of 7–7.5, which is indicative of a normal physiological pH and agrees with other glucosyltransferase (GT) pH optima. However, characterization of Cp3GT expressed using *P. pastoris* with a C-terminal c-myc-6x His tag showed a higher optimal pH of 8.5–9. This suggests a possible tag effect or an effect related to physiological differences between the cell expression systems. Results testing recombinant Cp3GT expressed in *Pichia* with and without C-terminal tags showed a possible tag effect with regard to substrate preference and interactions with metals, but no apparent effect on enzymatic kinetics or pH optima.

## 1. Introduction

Plants produce secondary metabolites that impart a wide range of beneficial effects and increase overall survivability. Flavonoids belong to the phenolic class of secondary metabolites and comprise a diverse class of over 6000 compounds that assist with plant defense, taste, smell, and coloration of flowers [1]. They play a role in insect-pollinator interactions and have been shown to facilitate the symbiotic interaction of nitrogen-fixating bacteria with legumes [2,3,4]. Flavonoids have anti-oxidant capabilities and participate in countless physiological activities. They, in part, account for the nutritional potential of many fruits and vegetables and have been shown to reduce inflammation and protect the heart [5]. Numerous flavonoids have shown medicinal potential as drugs to fight cancer [6,7].

Unraveling flavonoid biosynthesis has led to the production of novel plant variants with unique appearance and taste [8]. Engineering unique metabolic pathways requires a finely tuned collection of cell systems, enzymes, and substrates that are reconstituted in vitro to produce a desired compound [9]. Engineered pathways can be made more productive by incorporating enzyme components optimized through mutational characterization studies [10]. Isolating an enzyme for study relies widely on cell expression lines that use vector encoded tags for identification and purification. Some tags are only a few amino acids in length and are placed either at the very beginning or end of an enzyme, yet it is possible they may affect the structure of the enzyme, causing a change in activity [11]. Some vectors come with a cleavage site that allows removing tags to obtain the native enzyme, however, many enzymes are characterized with tags intact with no account given as to whether they impact activity [12,13,14]. It is generally assumed that tags have no impact on enzyme activity, yet research has shown instances where activity is in fact affected [15,16,17,18]. Furthermore, many enzymes are subsequently crystallized for structure determination with tags intact. These tags are not always reported in Protein Data Base (PDB) entries, yet there is evidence that inclusion of tags during crystallization can both aid or hinder crystallization depending on the enzyme, as well as influencing expression [19,20,21,22].

*Citrus paradisi* flavonol-specific 3-O glucosyltransferase (Cp3GT) (Genbank Protein ID: ACS15351) was previously characterized and shown to catalyze the transfer of glucose to the flavonols quercetin and kaempferol [22]. Cp3GT also showed activity with the flavonols myricetin, fisetin, and gossypetin to a lesser extent [23,24]. Cp3GT showed exclusive activity with flavonols and is regioselective for the 3-OH position. Cp3GT was initially cloned into the pCD1 vector, a modified PET32 vector that includes N terminal thioredoxin/6x His tags and a thrombin cleavage site [23]. Concerns regarding expression of Cp3GT in *E. coli* and inclusion body formation prompted cloning into the pPicZa vector and transforming into *P. pastoris* [23]. A thrombin recognition sequence was inserted into the recombinant pPicZa vector upstream of the C terminal c-myc/6x His tags. This was necessary for cleaving the tags so that untagged and tagged Cp3GT could be compared.

Initial characterizations of pH optima for Cp3GT, obtained through expression in *E. coli*, indicated an optimal pH of 7–7.5, which is indicative of a normal physiological pH. This agrees with other flavonoid glucosyltransferase (GT) pH optima of 6.5–8 [25,26,27]. More recent characterizations of Cp3GT expressed using *P. pastoris*, however, showed a higher optimal pH of 8.5–9. This suggests either an effect due to the location and chemistry of the recombinant tags, or an effect related to physiological differences between the cell expression systems [24]. This research was designed to test the hypothesis that recombinant tags have an effect on Cp3GT activity with respect to pH optima, substrate preference, kinetic parameters, and interaction with various metals.

## 2. Results and Discussion

### 2.1. Effect of Tags on Optimal pH

To make a comparison regarding pH optima between tagged and untagged enzyme, Cp3GT activity was measured over a pH range of 5.5–9.5 using various buffers that overlapped in pH. Among plant GT’s, pH optima tend to align with physiological pH (7–7.5) but can vary as high as 9 and as low as 5.5 when expressed in recombinant systems [26,28,29,30]. A shift away from physiological pH could suggest that conditions surrounding the expression and preparation of the enzyme are influencing stability and activity, especially for enzymes such as plant GT’s that have structural similarity. It is unclear what conditions define this influence, yet such a shift in pH optima was observed when Cp3GT was assayed using different cell expression systems and different recombinant tags. Recombinant Cp3GT expressed in *E. coli* and the enzyme expressed in the *Pichia* system showed different pH optima. One hypothesis is that this could be due to the different chemistry and position of the tags.

The characterization of Cp3GT showed an optimal pH of 7–7.5 for the protein expressed in *E. coli* with N terminal thioredoxin/6x His tags [23]. The tendency for bacterial cell-expression systems to generate mis-folded recombinant proteins as a result of conformational stress has been well studied [31]. Thioredoxin is a thermally stable 12 kDa protein often used in *E. coli* expression systems to aid in solubility of the recombinant protein, and has furthermore been used as a stabilizing fusion partner for driving crystallization in some instances [32,33,34]. However, given its size, it is possible that it could impact conformational folding of Cp3GT. Analysis of *E. coli* expressed Cp3GT with and without the N-terminal thioredoxin.6x His tags showed no impact of these tags on enzyme activity [22].

**Figure 1:**
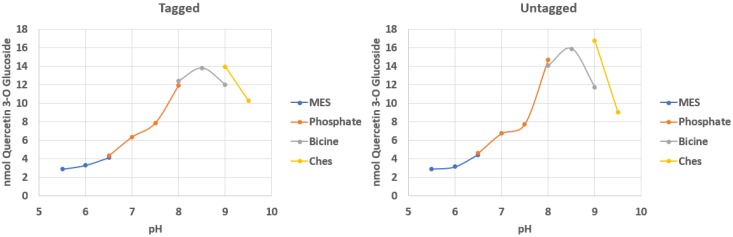
Optimal pH for activity in tagged and untagged Cp3GT from *P. pastoris*. Formation of quercetin 3-O glucoside was measured and plotted against the pH range tested. A mild buffer effect was seen with *N*-cyclohexyl-2-aminoethane sulfonic acid (CHES) pH 9. *n* = 2.

A characterization of pH optima was conducted for Cp3GT expressed in *P. pastoris* that contained C terminal c-myc/6x His tags. The optimal pH shifted to 8.5–9 in both tagged and untagged samples (Figure 1) which is higher than that observed for the enzyme expressed in *E. coli*. There also was an apparent CHES (*N*-cyclohexyl-2-aminoethane sulfonic acid ) buffer effect showing an increase in activity at pH 9 when compared with bicine. This is consistent with pH optima from a recent study reporting on mutational analysis of Cp3GT [24].

It was hypothesized that using different tags at the C terminus contributed to a higher pH optimum. However, cleaving the c-myc/6x His tag and testing untagged Cp3GT from *P. pastoris* over the same pH range showed no significant difference in activity levels between tagged and untagged Cp3GT (Figure 1). Likewise, cleaving the thioredoxin tag and testing untagged Cp3GT from *E. coli* over a pH range showed no significant difference in activity levels between tagged and untagged Cp3GT [22].

These findings together indicate no tag effects exist with regards to pH optima when Cp3GT is expressed in *P. pastoris* and contains a C terminal c-myc/6x His tag. This is consistent with data comparing tagged and untagged Cp3GT expressed in *E. coli*, wherein the presence of tags did not significantly alter pH stability [23]. It remains unclear why the optimum pH for Cp3GT activity shifted from 7–7.5 when expressed in *E. coli* to 8.5–9 when expressed in *Pichia pastoris*. While it may be something inherent to expression in prokaryotic versus eukaryotic systems, this remains to be determined. For example, it may be that Cp3GT may have been post-translationally modified in the yeast system, although this has not as yet been reported for plant GTs.

It should be noted that the overall yield of soluble Cp3GT enzyme was greater in the Pichia system. As previously stated, expression in the *E. coli* system resulted in the majority of Cp3GT being localized in inclusion bodies and the use of sonication to burst the cells to release soluble protein could have resulted in some denaturing of the protein. As a result, the amount of purified Cp3GT from *E. coli* needed to run kinetic reactions was over 50x greater than that used for the purified *Pichia* expressed protein (pH 7.5). The 0.5 µg Cp3GT from yeast used in our assays sustained linear velocity of activity for 10 min, compared with the 20–30 µg Cp3GT from *E. coli* that sustained linear velocity for 5 min. This suggests that a purer, cleaner enzyme is obtained when expressing Cp3GT in *P. pastoris*. The ability to obtain high concentrations of pure Cp3GT has positive implications for structure determination using X-ray crystallography.

### 2.2. Effect of Tags on Substrate Specificity of Cp3GT

**Figure 2:**
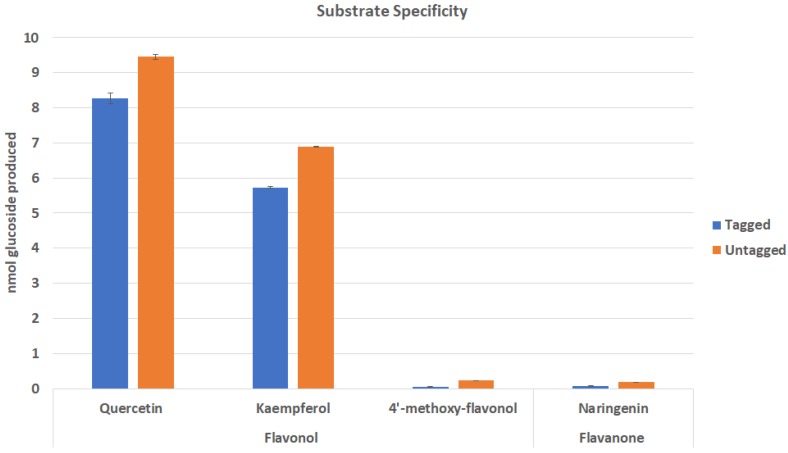
Effect of tags on Cp3GT substrate preference. Untagged Cp3GT showed greater activity with the preferred flavonol substrates quercetin and kaempferol. There was no significant activity with methoxylated flavonols or flavanones. *n* = 2.

Some studies have shown 6x-His tag interactions with catalytic structural features can impair substrate binding [18]. To investigate differences in substrate preference with regard to the presence or absence of recombinant tags, Cp3GT was assayed for activity with its preferred flavonol substrate quercetin, as well as two other flavonols (kaempferol and 4′-methoxy-flavonol), and the flavanone naringenin (Figure 2). This experiment tested the hypothesis that recombinant tags will have no impact on substrate preference.

In general, flavonol-specific Cp3GT shows the greatest activity with quercetin and kaempferol. It did not interact with flavonols containing methoxy groups, as seen by the lack of significant activity when assayed with 4′-methoxy-flavonol. Cp3GT was previously shown to be inactive when methoxy groups were placed on the flavonoid backbone [23,24,35]. There also was no significant activity with the flavanone naringenin which lacks a 3-OH group; this was also noted in previous studies [23,24,35]. The absence of tags appears to enhance Cp3GT activity with quercetin and kaempferol by approximately 14% and 20% respectively (Figure 2).

Other studies have shown that recombinant tags can impact activity in various ways [14,15,16,17]. One such interaction occurred between the catalytic binding site of a bacterial dehydrogenase and its recombinantly expressed C-terminal 6x His-tag that resulted in loss of activity due to alteration of the binding site [18]. In fact, the recombinant enzyme with an N-terminal tag showed 20-fold higher activity [18]. In silico analysis previously carried out on Cp3GT detected no interactions of this kind, however these models should be verified using X-ray crystallography [24]. With regard to substrate specificity of Cp3GT, it is possible that tags impact folding and conformation to some degree through structural interactions, and these interactions may alter substrate binding. The full scope of Cp3GT- recombinant tag interactions may be identified in future analyses using X-ray crystallography.

### 2.3. Effect of Tags on Kinetic Parameters

Kinetics assays were conducted to determine any tag effects related to K_m_^app^, V_max_, catalytic efficiency, and specificity constant. Kinetic assays were carried out under standard conditions of testing various concentrations of one substrate while having unlimiting concentrations of the other. Cp3GT exhibits Michaelis–Menten kinetics, thus the Lineweaver–Burke linear transformation was plotted to determine K_m_^app^ and Vmax (Table 1). This was done for Cp3GT containing the C-terminal tags and for Cp3GT with tags removed. No statistically significant differences were detected between tagged and untagged Cp3GT (mean ± SD). The K_m_^app^ for quercetin and kaempferol were not significantly different, though previous findings indicated a slightly higher K_m_^app^ for quercetin [23,24]. Furthermore, catalytic efficiency for quercetin was higher than kaempferol, which suggested more overall product could be produced when quercetin is used as acceptor substrate. These K_m_^app^ are similar to those found for the protein expressed from *E. coli* [22]. This would suggest that whatever is impacting the pH optima of the protein expressed in the two systems is not impacting the actual flavonol substrate kinetics.

The K_m_^app^ observed for UDP-glucose (Table 1) was approximately 10-fold lower than previously observed in Cp3GT expressed from both *E. coli* (669 µM) and *P. pastoris* (878 µM) [23,24]. Potential issues with amounts of active enzyme from the *E. coli* system were previously discussed. With respect to the current work, the Cp3GT protein was significantly purer than that in the previously published study on yeast expressed protein [24]. It is possible that while the protein was less active, it was still able to bind UDP-glucose thus making the K_m_^app^ appear significantly larger. For some enzymes that utilize UDP-glucose, K_m_^app^ values can be higher for the UDP donor substrate than for their corresponding acceptor substrate, yet still these values can vary considerably [36,37,38,39,40,41]. Vmax was noticeably lower for UDP-glucose than for acceptor substrates quercetin and kaempferol, and is lower than the previously reported values of 28.17 (Cp3GT from *E. coli*) and 83.5 (Cp3GT from *P. pastoris*) [23,24]. This could be due, in part, to the increased purity and activity of Cp3GT in this current study.

UDP has been shown previously to be a competitive inhibitor of Cp3GT as well as other flavonoid GT enzymes [23,35,42,43]. Inhibition was tested with tagged and untagged protein from the yeast expression system. Results again confirmed competitive inhibition with a Ki of 61.8 µM for tagged Cp3GT and a Ki of 73.1 µM for untagged Cp3GT. These results are comparable with the UDP Ki of 69.5 µM obtained during the first characterization of Cp3GT expressed in *E. coli* indicating that the presence of tags on the protein does not change the nature of competitive inhibition by UDP [23]. This suggests that the presence of N-terminal or C-terminal tags on Cp3GT does not impact competitive binding of UDP and the Kis are similar. This, coupled with similar K_m_^app^ for quercetin and kaempferol for tagged and untagged Cp3GT in this study, suggest that these tags do not significantly impact folding and orientation of the binding cleft.

#### Effect of Tags and Metals on Cp3GT Activity

Previous studies have shown that some metals can impact Cp3GT activity [23,42,44]. Therefore, nine metals were included in separate assays at concentrations of 1 and 10 mM (Table 2). Significant inhibition was observed for both tagged and untagged Cp3GT in reactions containing zinc, iron, calcium, copper, and manganese relative to control reactions without these compounds. A significantly greater inhibitory effect was observed for untagged samples using zinc and manganese for both 1 and 10 mM concentrations. Cp3GT activity was inhibited in samples containing both 1 and 10 mM iron with no significant difference between tagged and untagged samples. In the presence of 1 and 10 mM copper, both tagged and untagged Cp3GT were inhibited. Interestingly, magnesium significantly enhanced activity over control in the 10 mM sample, and the enhancement was greater in samples using untagged Cp3GT. KCl and NaCl also slightly enhanced Cp3GT activity.

Some differences were observed between tagged and untagged Cp3GT with regard to metal interactions. Specifically, untagged Cp3GT activity was enhanced in the presence of magnesium to a greater extent than tagged Cp3GT, however, in the presence of NaCl, tagged Cp3GT was enhanced to a greater extent over untagged Cp3GT. It remains unclear what specific interactions are driving this influence. It is possible that the stabilizing magnesium-UDP-G complex is more readily formed when magnesium is added to the solution. The need for magnesium in GT-catalyzed uridine diphosphate sugar transfer is well documented in biochemical systems, shown here by an increase in activity for samples containing added magnesium [45].

Consistent with previous findings, Cp3GT from *E. coli* and *P. pastoris* showed almost complete loss of activity in the presence of Fe^2+^, Cu^2+^, and Zn^2+^ ions [23,24]. Cp3GT from *E. coli* also showed a mild stimulatory effect in the presence of Mg^2+^ [23]. With respect to metal interactions, no differences were previously detected between tagged and untagged Cp3GT when expressed in *E. coli*.

## 3. Conclusions and Directions for Future Research

It was hypothesized that for pH optima, substrate binding, and interactions with inhibitor/activators, Cp3GT activity was impacted by the presence of a C-terminal c-myc/6x tag. Results do not support this hypothesis as this interaction does not appear to impact overall Cp3GT kinetics nor optimal pH. It is clear that recombinant tags do not explain the pH optima difference. Despite having no impact on kinetics or optimal pH, the differences observed in substrate binding and metal interaction between tagged and untagged Cp3GT suggest that recombinant tags effect substrate preference and metal binding. This could be resolved through structure determination by X-ray crystallography. Furthermore, it is possible that either donor or acceptor substrate more readily binds to untagged Cp3GT, thus aiding efforts to co-crystallize Cp3GT with substrate. These findings encourage the use of untagged Cp3GT when possible due to an apparent increase in substrate binding and activity over Cp3GT with tags intact.

While a few plant secondary product glucosyltransferases have had crystal structures solved [46,47,48,49,50,51], there is currently no crystal structure for a plant UDP glucosyltransferase that has the novel substrate and regiospecificity exhibited by Cp3GT. Additionally, there are no reports that compare crystal structures of plant UDP glucosyltransferases with and without tags. Therefore, future work to perform high-resolution structural analysis on Cp3GT will make a significant contribution to the field due to both its novel substrate and regiospecificity. It is also possible that this analysis will elucidate recombinant tag interactions that influence activity.

It is logical to propose that addition of tags to recombinant proteins could impede upon the determination of its true biological nature, yet unless gross deficiencies are observed experimentally, this assertion is often overlooked. Recombinant tag effects may only need to be investigated on a case by case basis, but it was indeed observed that Cp3GT binds flavonol substrates and interacts with some metals differently when a recombinant tag was affixed at the C terminus. Therefore, future work will focus on a high-resolution structural analysis of tagged Cp3GT that will elucidate both the structural basis for its novel specificity and determine how recombinant tags impact Cp3GT structure. Analysis of Cp3GT co-crystallized with acceptor and donor substrates would further elucidate both the nature of substrate binding itself, and whether recombinant tags effect substrate binding in some way. Structural analysis of Cp3GT will also include verifying recent models that predict residues responsible for Cp3GT substrate and regiospecificity. The effects of implementing additional purification steps to remove non-native artifacts is generally well tolerated in crystallizing GTs [52].

The ubiquity of recombinant tags in protein expression systems cannot be ignored and their use may almost be completely necessary. Indeed, their prevalence does not preclude thoroughly investigating the scope of recombinant tag effects. It is highly possible that determining the nature of these effects can greatly benefit the field.

## 4. Methods and Materials

### 4.1. Reagents and Materials

Reagents used were analytical grade and were obtained from the following sources: quercetin, FeSO_4_, CuSO_4_, and ZnCl_2_ were from MilliporeSigma (St. Louis, MO, USA); kaempferol was from Indofine (Hillsborough, NJ, USA), UDP-glucose was from Calbiochem (Gibbstown, NJ, USA), UDP- [U-14C] glucose was from PerkinElmer (Boston, MA, USA); Midiprep plasmid extraction kit was obtained from Promega (Madison, WI, USA). Miniprep plasmid extraction kit, MgCl_2_, MnCl_2_, KCl, and NaCl were purchased from Fisher Scientific (Waltham, MA, USA); CaCl_2_ was from Acros Organics (Morris Plains, Morris, NJ, USA); Na_2_SO_4_ was from Merk (Kenilworth, NJ, USA). Bovine thrombin was obtained from MP Biomedicals (Solon, OH, USA).

### 4.2. Insertion of Thrombin Cleavage Site and Transformation into Yeast

A thrombin cleavage site was cloned into the pPicZa plasmid containing the Cp3GT sequence (Genbank Protein ID: ACS15351) using Agilent’s QuikChange XL Site-Directed Mutagenesis Kit. Recombinant plasmid was transformed into competent *E. coli* cells and plasmid was sequenced to verify site insertion. DNA was transformed into X-33 *Pichia pastoris* of the Mut+ phenotype and expressed under methanol induction according to Invitrogen’s EasySelect Pichia Expression Kit and as previously described [24,35].

### 4.3. Removal of Recombinant Tags by Thrombin Digestion

Bovine thrombin was resuspended in 0.7% NaCl to a concentration of 20 U/µL. Approximately 2 units per microgram of Cp3GT was needed to achieve complete removal of tags at 4 °C for 2 h as verified by SDS-Page and Western Blot (Figures S1–S3).

### 4.4. Purification and GT Enzyme Assay

Extraction and purification of recombinant Cp3GT from yeast was carried out as previously described, using His-Pur immobilized cobalt metal affinity resin in gravity flow columns [22,34]. The bound resin was washed with 50 mL of sodium phosphate buffer containing 300 mM NaCl and 10 mM imidazole to remove non-specific, weakly bound proteins. Cp3GT was eluted using sodium phosphate buffer containing 150 mM imidazole. Eluent fractions were pooled, dialyzed, and concentrated into 50 mM NaPO4 buffer, pH 7.5 containing 14 mM BME using Amicon-15 (30 kDa) centrifugal filters. Purification was confirmed through SDS-Page and Western Blot. Enzyme activity assays were conducted as previously described using 0.5 µg of purified protein at 30 °C, pH 7.5. [42]. Initial time course assays indicated reaction was linear for at least 10 min, thus all reactions were run for 10 min.

#### 4.4.1. pH Optima

For enzymatic assays testing optimal pH, reactions were buffered with either 2-ethansulfonic acid (MES, pH 5.5, 6, and 6.5), potassium phosphate (pH 6.5, 7, 7.5, and 8), bicine (pH 8, 8.5, and 9), or CHES (pH 9 and 9.5). Reactions were carried out as previously described using 0.5 µg of purified Cp3GT at 30 °C, pH 7.5.

#### 4.4.2. Substrate Specificity

Enzymatic assays testing substrate specificity were carried out as previously described using 0.5 µg of purified Cp3GT at 30 °C, pH 7.5. Each reaction contained 50 nanomoles of either quercetin, kaempferol, 4′-methoxy-flavonol, or naringenin.

#### 4.4.3. Kinetics

Assays measuring Cp3GT kinetics were carried out using increasing concentrations of quercetin and kaempferol ranging from 0–667 µM. Kinetic assays using UDP-G were carried out using increasing concentrations ranging from 0–1333 µM. All reactions were carried out as previously described using 0.5 µg of purified Cp3GT at 30 °C, pH 7.5.

#### 4.4.4. Metals and UDP Inhibition

Enzymatic assays testing metal interactions were carried out as previously described. Each reaction contained one of nine metals at a final concentration of either 1 or 10 mM. For assays measuring UDP inhibition, reactions contained either 0, 0.05, or 0.1 mM UDP.

### 4.5. Statistial Analysis

Calculations were performed to determine mean ± SD for replicate experiments. Sample results with overlapping SD bars were considered not significantly different.

## Figures and Tables

**Table 1:** Kinetics on tagged and untagged Cp3GT with three substrates. Values are expressed as mean ± SD. *Average of 2 experiments (*n* = 2) **Average of 6 experiments (*n* = 2). UDPG = Uridine Diphosphoglucose.

Substrate		K_m_^app^ (µ)	V_max_ (pKat/µg)	K_cat_ (s^−1^)	K_cat_/K_m_^app^ (µM/s)
Quercetin*	Tagged	51.9 ± 8.29	31.7 ± 3.39	1.84 ± 0.19	0.035 ± 0.001
	Untagged	42.4 ± 3.48	26.5 ± 1.06	1.54 ± 0.06	0.036 ± 0.001
Kaempferol*	Tagged	44.1 ± 3.41	22.0 ± 0.56	1.28 ± 0.02	0.029 ± 0.001
	Untagged	53.7 ± 10.6	23.1 ± 3.00	1.34 ± 0.17	0.025 ± 0.001
UDPG**	Tagged	49.7 ± 5.32	10.9 ± 1.59	0.637 ± 0.092	0.012 ± 0.002
	Untagged	55.0 ± 4.97	12.7 ± 2.15	0.738 ± 0.125	0.012 ± 0.0004

**Table 2:** Activity of Cp3GT in the presence of various metals. Of the metals tested, ZnCl_2_, NaCl, MnCl_2_, MgCl_2_ were significantly different between tagged and untagged Cp3GT. Values are expressed in % relative activity (*n* = 2).

	% Relative Activity			
Metal	Tagged		Untagged	
	1 mM	10 mM	1 mM	10 mM
Untreated	100	100	100	100
ZnCl_2_	44	28	25	23
KCl	117	119	114	112
FeSO_4_	42	16	42	5
NaCl	118	121	100	95
Na_2_SO_4_	92	88	92	87
CaCl_2_	89	69	84	72
CuSO_4_	79	3	70	3
MnCl_2_	87	87	60	56
MgCl_2_	104	123	103	140

## Supplementary Materials

The following are available online at https://www.mdpi.com/2223-7747/9/3/402/s1, **Figure S1: Cp3GT Digest with Increasing Amounts of Thrombin.** Complete removal of tags indicated by absence of bands. The lack of a band is due to the removal of the c-myc peptide that corresponds to our antibody. **Figure S2: Coomassie Stain of Cp3GT Before and After Treating with Thrombin.** Cp3GT can be seen at 56 kDa in its native form, whereas with tags it runs slightly higher due to the increase in size from the recombinant tags. Thrombin is shown at 36kDa in untagged samples. **Figure S3: Western Blot of Cp3GT Before and After Treating with Thrombin.** Complete removal of tags indicated by the absence of a band in the untagged sample.
