# Supplementary material for: The Effect of Recombinant Tags on Citrus paradisi Flavonol-Specific 3-O Glucosyltransferase Activity
Source: Plants (Basel). 2020 Mar 24;9(3):402. doi: 10.3390/plants9030402 (PMC7154896; doi:10.3390/plants9030402)
Supplement: Supplementary file 1 [file plants-09-00402-s001.pdf]

plants

MDPI

Supplemental Materials

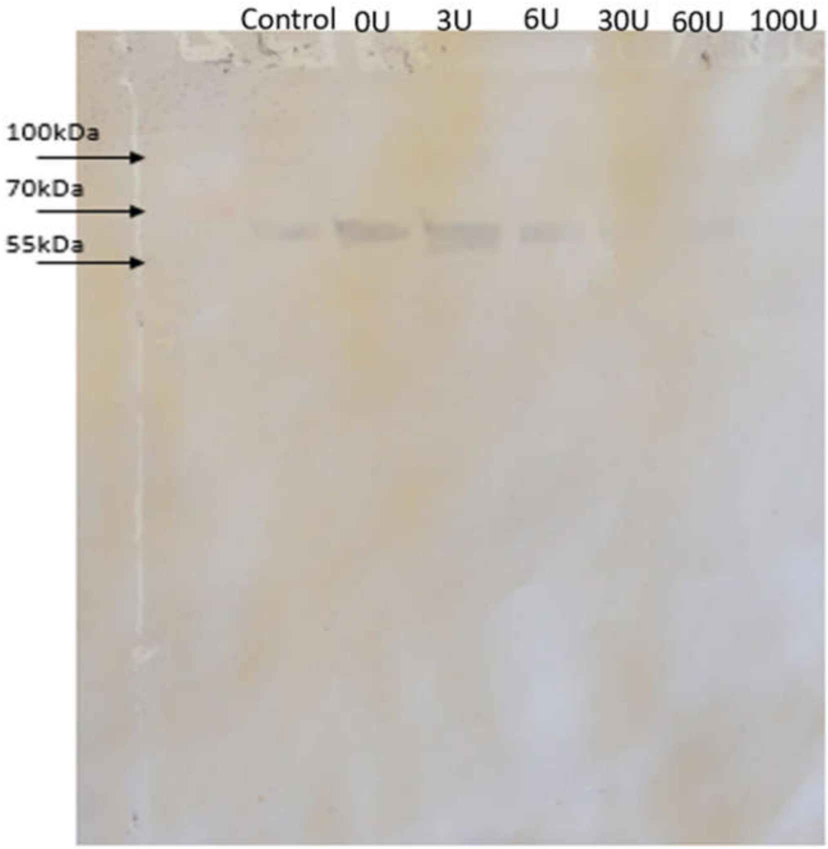

Figure S1: Cp3GT Digest with Increasing Amounts of Thrombin. Complete removal of tags indicated by absence of bands. The lack of a band is due to the removal of the c-myc peptide that corresponds to our antibody.

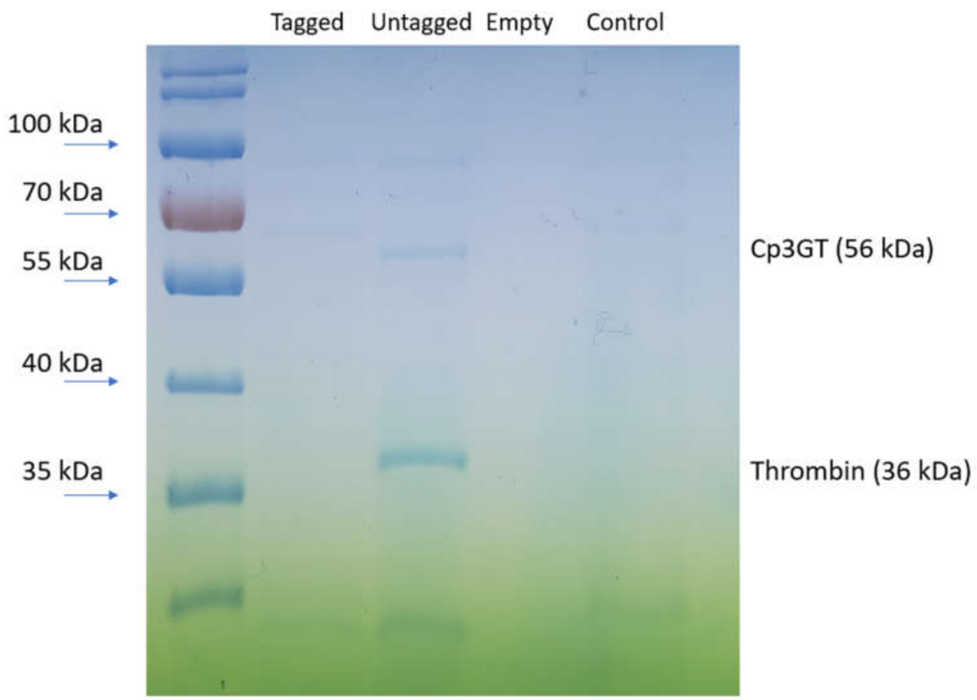

Figure S2: Coomassie Stain of Cp3GT Before and After Treating with Thrombin. Cp3GT can be seen at 56 kDa in its native form, whereas with tags it runs slightly higher due to the increase in size from the recombinant tags. Thrombin is shown at 36kDa in untagged samples.

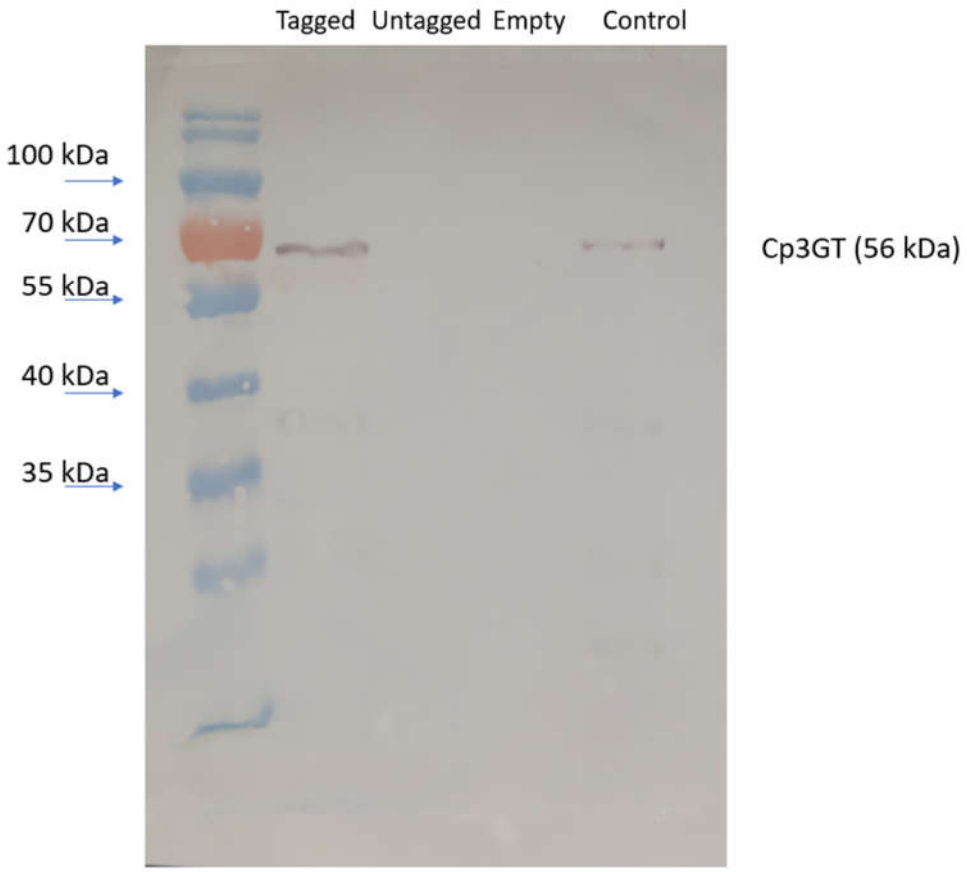

Figure S3: Western Blot of Cp3GT Before and After Treating with Thrombin. Complete removal of tags indicated by the absence of a band in the untagged sample.
